# EBV^+^ and MSI Gastric Cancers Harbor High PD-L1/PD-1 Expression and High CD8^+^ Intratumoral Lymphocytes

**DOI:** 10.3390/cancers10040102

**Published:** 2018-04-01

**Authors:** Simona De Rosa, Nora Sahnane, Maria Grazia Tibiletti, Francesca Magnoli, Alessandro Vanoli, Fausto Sessa, Anna Maria Chiaravalli

**Affiliations:** 1 Department of Pathology, Ospedale di Circolo, 21100 Varese, Italy; simona-derosa@hotmail.com (S.D.R.); mariagrazia.tibiletti@asst-settelaghi.it (M.G.T.); francesca.magnoli@asst-settelaghi.it (F.M.); fausto.sessa@uninsubria.it (F.S.); 2 Department of Surgical and Medical Sciences, University of Insubria, 21100 Varese, Italy; n.sahnane@gmail.com; 3 Department of Molecular Medicine, University of Pavia and IRCCS S. Matteo Hospital, Pavia 27100, Italy; alessandro.vanoli@unipv.it

**Keywords:** gastric cancer, PD-L1, EBV, MSI, tumor infiltrating lymphocytes

## Abstract

Both EBV^+^ and MSI gastric cancers (GCs) have high lymphoid infiltration which is rare in MSS/EBV^−^ cancers. PD-L1/PD-1 interaction leads to a down-regulated immune response and it is one of the most promising targets for gastric cancer immunotherapy. PD-L1/PD-1 and CD8 expression were immunohistochemically investigated in a series of 169 FFPE GCs, including 33 EBV^+^, 59 MSI and 77 MSS/EBV^−^ cases. PD-L1 membrane immunoreactivity in more than 5% of tumor cells was present in 31/169 GCs and was associated with high levels of CD8 intraepithelial lymphocytes (TILs; *p* < 0.001). PD-L1^+^ cases were mainly poorly differentiated (71%), intestinal type (85%) and high lymphoid response (HLR; 90%) tumors. PD-L1 expression was only present in EBV⁺ (46%), MSI (24%) and rare MSS/EBV^−^ (3%) GCs with high CD8^+^ TILs (*p* < 0.001). Despite being associated with a better prognosis both in the whole series (*p* < 0.05) and in the MSI subset, PD-L1 is not an independent prognostic factor. PD-L1 gene amplification was detected in 3/17 cases, including 2/7 EBV^+^ and 1/8 MSI GC. PD-1⁺ TILs were significantly higher in EBV⁺ than MSI and MSS/EBV^−^ cases. PD-L1/PD-1 pathway is selectively activated in HLR GCs and could be considered an emerging therapeutic target, particularly for EBV and MSI GCs.

## 1. Introduction

Gastric cancer (GC) is a heterogeneous disease characterized by different molecular and histological profiles [1,2], which are associated with different grades of immune responses. A high lymphoid infiltration is a peculiar feature of microsatellite instable (MSI) and EBV infected (EBV^+^) GCs. 

As in other malignancies such as melanoma and non-small cell lung cancer, GC tumor cells can also activate immune checkpoints to inhibit and escape an immune system response. One of these checkpoints is programmed cell death-ligand 1/programmed cell death protein-1 (PD-L1/PD-1) interaction. PD-L1 (B7-H1/CD274) is a type I transmembrane glycoprotein constitutively expressed on macrophages, T, B and dendritic cells, and its expression is enhanced by pro-inflammatory cytokines. In normal tissue, PD-L1 protein is less synthesized compared to its mRNA which is present in various human tissues [3]. PD-1 is a type I transmembrane glycoprotein receptor expressed on mature B- and T-cells upon activation and on CD4⁻/CD8⁻ thymocytes in transition to CD4⁺/CD8⁺ stage. PD-1 expression is up-regulated after interaction between T-lymphocytes and a specific antigen and decreases according to antigen elimination [3].

In cancers, up-regulation of PD-L1 expression could depend on intrinsic cellular control directed by genomic alterations, or it could be the consequence of an adaptive extrinsic control induced by CD8^+^ T cells [4]. In addition to colorectal cancer, melanoma, renal cell carcinoma and non-small cell lung cancer, PD-L1 expression has been described on tumor cells also in GCs, where it has been demonstrated to be associated with EBV infection or MSI status or with a massive T lymphocyte presence [5,6,7,8,9]. 

The role of PD-L1 expression as a prognostic factor remains unclear because of the absence of uniform evaluating criteria for its analysis and the different tumor characteristics analyzed and correlated to survival in the various studies [5,10,11,12,13].

In this work we evaluated PD-L1/PD-1 expression and CD8^+^ tumor infiltrating lymphocytes (TILs) in whole formalin-fixed, paraffin-embedded tissue sections of a large series of 169 GCs which were well characterized histologically and enriched for EBV^+^ and MSI cases. The role of PD-L1 in predicting survival was also investigated. 

## 2. Results

### 2.1. PD-L1 Expression

PD-L1 immunoreactivity was observed in tumor cells and in immune cells, lymphocytes and macrophages infiltrating and surrounding tumor nests. 

PD-L1^+^ tumor cells, varying from 1% to 90%, showed weak to strong immunoreactivity prevalently along the cellular membrane or both along the membrane and in the cytoplasm. Five cases displayed only cytoplasm immunoreactivity and were considered negative for PD-L1.

Thirty-one out of 169 (18.3%) GCs were PD-L1^+^, showing 5% or more PD-L1^+^ tumor cells. They were mainly poorly differentiated GCs, intestinal in type according to the Lauren Classification, with the extent of the primary lesion (pT) being low and a prevalence of TNM stage I and II (Table 1). They occurred more frequently in male (58%) with significantly older age and displayed a larger diameter than PD-L1 negative GCs.

According to the histotype-based prognostic classification [14], 28/31 (90%) PD-L1^+^ cases were high lymphoid response (HLR) GCs, 2 were ordinary cohesive (one MSI GC with a moderate lymphoid reaction, the other a MSS/EBV^−^ GC with only one tumor area rich in lymphocytes) and one was an anaplastic MSI GC with a moderate number of TILs. Noteworthy, no ordinary or invasive mucinous carcinomas, including MSI cases, showed PD-L1 expression.

PD-L1^+^ cases were significantly more frequent among EBV^+^ (15/33, 46%) and MSI (14/59, 24%) than in MSS/EBV^−^ (2/77, 3%) GCs (*p* < 0.001). When we considered the cut-off of 1%, instead of 5% of immunoreactive tumor cells, the percentage of PD-L1^+^ cases increased to 61% for EBV^+^ and 37% for MSI GCs, whereas it remained unvaried in MSS/EBV^−^ GCs. 

**Figure 1:**
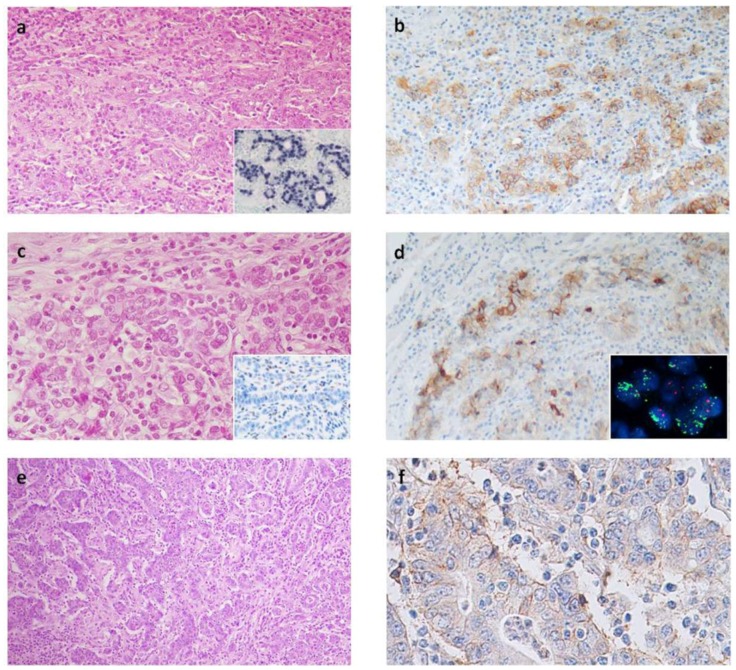
(**a**) EBV^+^ gastric carcinoma showing lymphoid stroma and (**b**) abundant PD-L1+ tumor cells spread out along the tumor; (**a inset**), in situ hybridization for EBV RNA, EBER; (**c**) The MSI gastric cancer lacking MSH2 protein (**c inset**); with (**d**) intense PD-L1 expression prevalently along the infiltration front and (**d inset**) high levels of PD-L1 gene amplification; (**e**) One of the MSS/EBV^−^ gastric carcinoma showing abundant lymphocytes infiltration and (**f**) weak PD-L1 immunoreactivity along membrane of tumor cells. Original magnification: a, b, d, e, a inset and c inset, 100×; c, 200×; f, 400×; d inset, 1000×.

In EBV^+^ cases, PD-L1^+^ tumor cells were spread out along the tumor and varied from 1% to 90% (mean % of immunoreactive cells in positive cases: 33%; Table 2). MSI GCs were characterized by a lower percentage of tumor immunoreactive cells, varying from 1% to 40% (mean value in positive cases: 18%) and prevalently localized along the tumor infiltration front (Figure 1). 

Among MSS/EBV^−^ cases a weak PD-L1 immunoreactivity was observed in only two cases, in 15% and 20% of tumor cells, respectively (Figure 1). Histologically, one case was an HLR GC, the other one was a cohesive carcinoma with a tumor component rich in lymphocytes.

In addition to PD-L1^+^ tumor cells, moderate to strong PD-L1^+^ immune cells were present in 94% (31/33 cases) of EBV^+^ and 37% (22/59 cases) of MSI GCs. Regarding the pattern of infiltration, in addition to the invasive margin the PD-L1^+^ immune cells infiltrated tumor in 58% and 54% of EBV^+^ and MSI GCs, respectively (TI, Table 2). On the contrary, in MSS/EBV^−^ GCs PD-L1^+^ immune cells were observed in only 25% (19/77, *p* < 0.001) of the cases. They were few and prevalently localized along the invasive margin (IM). TI pattern, in fact, was present in only 1/19 (95%, *p* < 0.001).

### 2.2. CD8^+^ Lymphocytes

CD8^+^ lymphocytes were present both within tumor-cell nests and in intratumoral and peritumoral stroma. The mean number of CD8^+^ TILs varied from 0 to 167 and was significantly (*p* < 0.001) higher in EBV^+^ (64.2; range 14.4–167) and MSI (23.1; range 0–69.2) GCs than in MSS/EBV^−^ tumors (15.8; range 0–94.4, Table 2).

Considering the previously defined cut-off value of 9.5 CD8^+^ TILs for HPF [15], PD-L1^+^ cases were significantly associated with the presence of high levels of CD8^+^ TILs (29/107, 27% GCs with high CD8^+^ TILs vs. only 2/48 (4%) GCs with low CD8^+^ TILs, *p* < 0.001; Table 1).

### 2.3. PD-1^+^ Intraepithelial Lymphocytes

The presence of PD-1^+^ TILs was observed in 137/169 GCs (81%), more frequently in EBV^+^ (30/33, 91%) and MSI (51/59, 86%) than MSS/EBV^−^ (56/77, 73%; *p* < 0.05) GCs (Table 2). Overall, the mean value of PD-1^+^TILs was 7 cells/HPF; EBV⁺ GCs were characterized by a significant higher value of PD-1^+^ cells (17.5 cells/HPF) compared to MSI (7 cells/HPF) and MSS/EBV⁻ (3.1 cells/HPF; *p* = 0.01) GCs (Table 2).

### 2.4. FISH Results

FISH analysis was performed in 17 cases (7 EBV^+^, 8 MSI and 2 MSS/EBV⁻ GCs), including 12 high PD-L1 expression and 5 PD-L1 negative GCs with only cytoplasm immunoreactivity. The probe used for this study identified CD274,PDCD1LG2 gene cluster amplifications; high levels of amplification of this region were observed in three out of 12 PD-L1^+^ cases (Figure 1d, inset). All amplified GCs revealed a gene copy number greater than 10 (ratio more than 5) in diploid cells, while polisomy of chromosome 9 without amplification was observed in nine cases. The amplified cases were two EBV^+^ with 90% and 40% PD-L1^+^ cells and one MSI GCs, defective for MSH2 and MSH6 proteins, and showing 30% PD-L1^+^ cells. None of the 5 cases case with only high cytoplasm PD-L1 immunoreactivity was amplified.

### 2.5. Survival Analysis

Because of the very low mortality rate in early (pT1) gastric cancers, correlations with survival were analyzed only for advanced (pT2, pT3 and pT4) GCs (AGCs). Follow up data were available for 146 AGCs. According to the literature, at univariate analysis intestinal (*p* = 0.002) and HLR (*p* < 0.0001) histotypes, low TNM stage (*p* < 0.0001), low pT (*p* < 0.0001), absence of lymph node metastases (*p* = 0.002) and high CD8 TILs (*p* = 0.01) were significantly correlated with a better outcome (Table 3).

**Figure 2:**
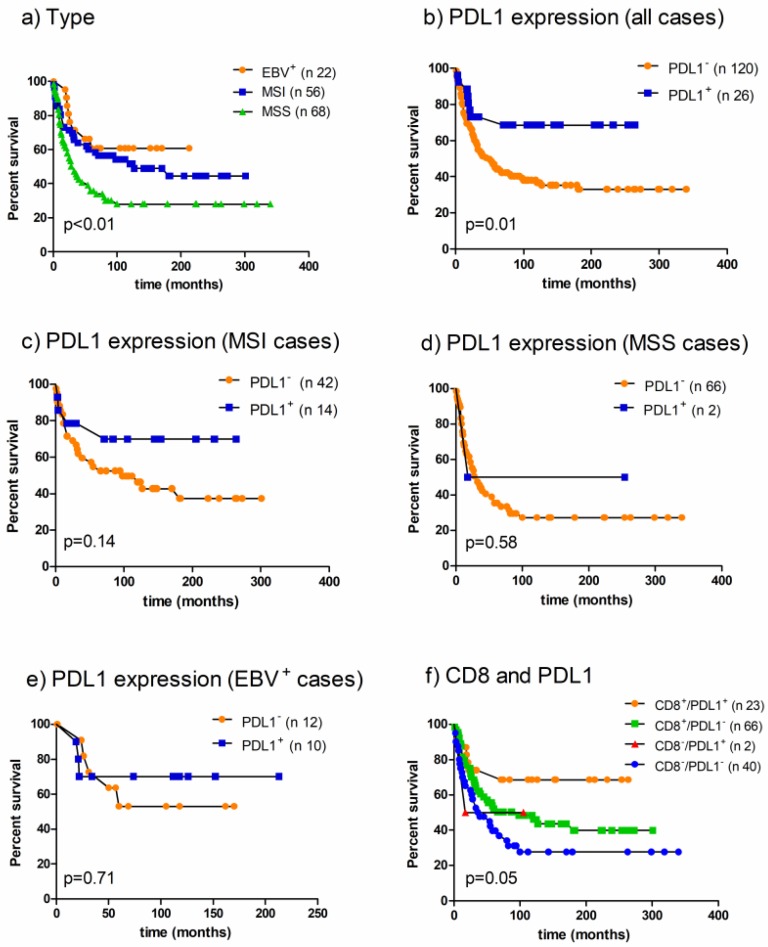
Failure time according to (**a**) EBV and MSI status (log-rank chi-square 9.34); (**b**) PD-L1 expression in the whole series (relative risk 2.44, log-rank chi-square 6.21); (**c**) PD-L1 expression in MSI GCs (relative risk 2.15, log-rank chi-square 2.13); (**d**) PD-L1 expression in MSS/EBV^−^ GCs (relative risk 0.30, log-rank chi-square 0.58); (**e**) PD-L1 expression in EBV^+^ GCs (relative risk 1.31, log-rank chi-square 0.13 *p* = 0.71) (**f**) and PD-L1 expression and CD8^+^ TILs (log-rank chi-square 7.75).

In addition, cancer-specific survival was better for EBV^+^ and MSI AGCs (*p* = 0.009, Figure 2a) and, accordingly, for PD-L1^+^ cases (*p* = 0.01, Figure 2b). To better understand the influence of PD-L1 expression on survival the analysis was performed for EBV^+^, MSI and MSS/EBV GCs separately. PD-L1 expression resulted in being associated with a more favorable behavior in the MSI (Figure 2c), but not in the MSS/EBV^−^ (Figure 2d) and EBV^+^ (Figure 2e) subset.

The cases were stratified in 4 groups according to the presence or absence of PD-L1 expression and high or low levels of CD8^+^ TILs. GCs with PD-L1 expression and high amounts of CD8^+^ TILs were characterized by a better prognosis (*p* = 0.05; Figure 2f).

Based on a Cox regression analysis, only a low tumor stage (*p* < 0.0005) and high number of CD8^+^ TILs (*p* < 0.05) or HLR histotype (*p* < 0.05) were identified as independent prognostic factors (Table 4). 

No statistically significant correlation was observed between PD-1 expression and survival.

## 3. Discussion

EBV^+^ and MSI GCs account for about 20% of GCs and show a similar histological pattern characterized by a high intratumoral lymphocyte infiltration, prevalently composed of activated cytotoxic CD8^+^ cells, as previously demonstrated [15]. The host immune response against tumors, induced by the production of abnormal peptides by the virus or by a defective mismatch repair system, can play a pivotal role in the more favorable outcome observed in these tumors, but it is not enough to prevent tumor growth. The reason tumors with an activated immune system escape the immune surveillance may be explained by the upregulation of mechanisms of immune tolerance. The binding of PD-L1 to its receptor PD-1 reduces effector T-cell function suppressing cytotoxic production, proliferation, and migration of T cells [16,17]. To our knowledge the first report demonstrating PD-L1 expression in GCs was published in 2006 [18] and in the last few years several studies have confirmed these data and better analyzed the PD-L1/PD-1 pathway in GCs. According to the literature, we demonstrated that GCs show PD-1^+^ immune cells and PD-L1 expression, both in tumor and immune cells [5,7,8,9,10,11,12,19,20]. In positive cases, PD-L1 immunoreactive tumor cells were heterogeneously distributed among tumor sections, mainly reflecting the distribution of lymphocyte infiltration. By counting CD8^+^ cells in direct contact with tumor cells, both within tumor nests and between tumor cells and stroma along the invasive border, PD-L1 positive cases were significantly associated with high CD8 TILs (more than 9.5 CD8^+^ cells/HPF). 

The majority of PD-L1^+^ cases belong to the HLR group [14], which is characterized by high levels of CD8^+^ T-cells infiltrating tumoral epithelium and stroma and that includes EBV^+^, MSI and a small number of GCs with high lymphoid infiltration but absence of virus infection and a defective MMR system. In our series about 50% of EBV^+^ and 25% of MSI GCs expressed PD-L1 in more than 5% of tumor cells. Outside these two distinct subgroups PD-L1 was observed in only two (3%) other cases, both MSS/EBV^−^ GCs characterized by a high lymphoid infiltration that affects the whole tumor area or only a part of it. In addition, EBV^+^ cases showed a higher number of PD-L1^+^ cells, CD8^+^ TILs and PD-1^+^ cells than MSI and both compared to MSS/EBV^−^ GCs. By confirming previous observations on smaller series of GCs [8,19], PD-L1^+^ IC were also significantly more frequent in EBV^+^ and MSI than MSS/EBV^−^ cases. In the last subset of carcinomas, positive IC, if present, were confined at the invasive front, whereas they infiltrate tumor in EBV^+^ and MSI GCs. 

Since PD-L1^+^ GCs were prevalently EBV^+^ or MSI, their clinic-pathological profile is a mixture of both EBV and MSI characteristics. Furthermore, our GCs do not belong to a consecutive series of cases, but rather are enriched in both EBV and MSI carcinomas to compare them to MSS/EBV^−^ subgroup. Therefore, the frequency of some features may be altered by such enrichment. The high frequency of low TNM stages in PD-L1^+^ cases, for instance, could be more likely related to the presence of early GCs (pT1) in EBV, but not in MSS/EBV^−^ group, rather than to PD-1/PD-L1 pathway activation. Anyway, PD-L1^+^ carcinomas were prevalently poorly differentiated intestinal GCs, with larger diameter, lower pT and stage, with a preference for male and older patients. As previously observed by Ma and colleagues [19] all mucinous carcinomas were negative for PD-L1, suggesting that mucus secreted by tumor cells can hide them from immune system. The major peculiar feature of PD-L1^+^ GCs remains the presence of high CD8^+^ lymphocytes. 

Previous papers reported the association between PD-L1 expression and CD8^+^ T-cells infiltration [7,9,20], both in gastric and other malignancies. PD-L1 upregulation on tumor cells may be the manifestation of an adaptive immune resistance induced by CD8^+^ lymphocytes via γ interferon secretion or by an intrinsic resistance due to oncogenic signaling. Derks et al. [8] observed that EBV and MSI GCs have a high INF-γ gene expression response with respect to MSS/EBV^−^ GCs. In a recent paper, Mimura and colleagues [21] demonstrated a significantly increased PD-L1 expression, mainly via the JAK-STAT pathway, in gastric cancer cell lines treated with IFN-γ and a significant positive correlation between PD-L1 expression on tumor cells and high levels of stromal CD8^+^ T cells and tumor IFN-γ. All these data seem to suggest that an adaptive immune resistance contributes to PD-L1 expression rather than an innate immune resistance.

On the other hand, the TCGA research network [1] reported 9p24.1 amplification and increased PD-L1 mRNA expression in two out 12 EBV^+^ cases. Also, Clavè and colleagues [22] described the presence of 9p24.1 amplification resulting in high PD-L1 expression in 8% of lung cancers. We observed that some of our PD-L1^+^ GCs showed a very strong immunoreaction involving a high number of tumor cells. FISH analysis demonstrated PD-L1 gene amplification in 3 out of these cases, which were two EBV^+^ and one MSI GCs. The induction of PD-L1 expression by inflammatory cytokines may be more efficient in tumors with gene amplification and the knowledge of the presence of an oncogenic upregulation of PD-L1, in addition to an adaptive immune response, could be useful for choosing the appropriate immunotherapy.

Controversial data are reported in the literature about the impact of PD-L1 expression on survival in GCs [5,7,12,13,19,20]. The discrepancies could be due to different factors, such as the antibody and the cut-off value adopted, the use of tissue microarrays instead of whole tissue sections in a so heterogeneous pattern of immunoreactivity, the choice of consecutive or selected series, and above all, the parameters included in the multivariate analysis. In our whole series, after excluding early GCs, PD-L1 expression was associated with a better prognosis and the data is not surprising since its expression is higher in MSI and EBV^+^ GCs and is associated with high levels of CD8^+^ TILs, all parameters related to a more favorable prognosis. Interestingly, subgroup analyses demonstrated that cancer-specific survival was better for PD-L1^+^ cases in the MSI but not in the EBV^+^ subgroup. It could be due to the low number of EBV^+^ advanced GCs or the significantly higher presence of PD-1^+^ lymphocytes in EBV^+^ than MSI GCs. A more favorable prognosis was also observed when PD-L1 expression was combined with high CD8^+^ TILs. This result might be explained again by the CD8^+^ TILs positive influence on prognosis. Multivariate analysis, however, showed that PD-L1 expression is not an independent prognostic factor. Tumor stage and HLR histological type or high levels of CD8^+^ lymphocytes are the only independent prognostic parameters, stronger than MSI and EBV status.

In conclusion, PD-L1/PD-1 expression is a distinctive biological feature of EBV^+^, MSI and rare MSS/EBV^−^ gastric cancers characterized by abundant CD8^+^ lymphocyte infiltration. In these tumors the use of immune checkpoint inhibition can restore the efficiency of immune system. The presence of PD-L1 gene amplification can contribute to PD-L1 upregulation. 

Low TNM stage and high CD8^+^ TILs, but not PD-L1 expression, are independent positive prognostic factors.

## 4. Materials and Methods 

### 4.1. Patients and Samples

The study was performed on 169 cases of advanced and early gastric carcinomas (GCs) operated on at the Ospedale di Circolo Fondazione Macchi in Varese and Fondazione Policlinico San Matteo in Pavia (Italy) between 1980 and 2012. Patients who had undergone preoperative chemotherapy or radiotherapy were excluded from the study.

Most of the cases belong to the Varese series of GCs, previously well characterized both histologically and for the presence of EBV infection, microsatellite instability (MSI) and MMR protein expression [14]. Some EBV positive (EBV^+^) and MSI cases had to be excluded because there was no remaining tumor tissue; thus, to make the groups comparable, new cases were added from the Pavia series. Altogether, 33 cases where EBV^+^, 59 were MSI and 77 were microsatellite stable-EBV negative (MSS/EBV^−^) carcinomas.

The tumors were classified according to the Lauren criteria [23] and histotype-based prognostic classification criteria previously described [14]. The disease stage was assessed according to the TNM criteria, 7th edition [24].

Patients were followed-up either until death or for a mean period of 164 months (range 3–340).

The clinico-pathological data of the GCs are summarized in Table 1.

The study was performed in accord with the ethical standards established by the Local Institution Review Board, and in accord with the Helsinki Declaration of 1975.

### 4.2. Immunohistochemical Study

Immunohistochemical reactions for PD-L1, PD-1 and CD8 were performed on whole tissue sections obtained from formalin-fixed, paraffin-embedded tumor blocks. Three μm thick sections were deparaffinized and rehydrated. Endogenous peroxidase activity was inhibited in 3% H_2_O_2_ in water for 15 min. Slides were treated for antigen-retrieval with pH8 EDTA buffer in a domestic microwave oven for 20 min. 

Primary antibodies were applied overnight at −4 °C. In particular, PD-L1 rabbit monoclonal antibody (clone SP142, Spring Bioscience, CA, USA) was used at a working dilution of 1/100; PD-1 mouse monoclonal antibody, (clone NAT 105, Ventana Medical Systems, AZ, USA) was undiluted, while CD8 monoclonal antibody (clone 4B11, Novocastra New Castle, UK) was used at a dilution of 1/20. 

UltraVision Quanto Detection System HRP (Thermo Scientific, Lab Vision Corporation, Fremont, CA, USA) was used as amplification system according to the manufacturer’s instructions and with 3-3′-Diaminobenzidine as the chromogen. 

PD-L1 immunoreactivity was evaluated separately in tumor and immune cells. For tumor cells, only membrane staining was considered, and the percentage of positive cells was recorded, independently of the intensity. A case was considered positive if PD-L1 membrane immunostaining was present in at least 5% of tumor cells. For immune cells, both the pattern of infiltration and the intensity of immunoreaction were evaluated. The intensity was scored as absent/weak, moderate or strong, whereas PD-L1^+^ immune cells were recorded as tumor infiltrating (TI) or located only along the invasive margin (IM), as reported previously [8]. PD-1^+^ and CD8 TILs were counted at 400× (Leitz, Laborlux K; field area 0.173 mm^2^; Leica Microsystems GmbH, Wetzlar, Germany) in ten consecutive fields and the mean value of immunoreactive TILs per high power fields was reported. The count area was selected according to the maximal number of neoplastic cells with minimal desmoplastic stroma and necrosis and only the immunoreactive lymphocytes in direct contact with tumor cells were included in the count.

### 4.3. Fluorescence In Situ Hybridization (FISH) Analysis

All the cases showing intense and abundant (more than 30% of tumor cells) membrane or cytoplasmic immunoreactivity for PD-L1 were tested for the presence of gene amplification. Interphasic FISH analysis was performed on 3–4 μm-thick sections used for conventional histologic examination as reported in the guidelines of the European Cytogeneticists Association [25] and the cytogenetic interpretation of data agrees with the International System for human Cytogenetic Nomenclature ISCN [26]. FISH analysis was performed using direct viewing on a standard fluorescence microscope at ×100 magnification. FISH results were evaluated on representative areas of each tumor identified on hematoxylin and eosin stained slides. To ensure a representative sample and to permit an assessment of the extent of tumor heterogeneity, CD274,PDCD1LG2 region amplification and chromosome 9 polisomy were scored in more than 200 interphasic nuclei from different separate areas of each tumor. Only experiments with 90% hybridization efficiency were considered. PDL1 amplification was investigated simultaneously hybridizing CD274,PDCD1LG2 region (green signal) and the centromere of chromosome 9 (red signal) with Zytolight SPEC CD274,PDCD1LG2/CEN 9 Dual Color Probe, (Zytovision GmbH, Bremerhaven, Germany). Cases were defined as amplified when the ratio (R) between green (CD274,PDCD1LG2) and red (CEN9) signals was >2.0. Cases were defined as polysomic for chromosome 9 when at least 20% of neoplastic cells showed three or more copies of CEN9 signals (red signals).

### 4.4. Statistical Analysis

Statistical analysis was performed by using Fisher’s exact test or Chi-square test and Wilcoxon rank-sum test for unpaired data. Survival curves were calculated using the Kaplan-Meier estimator test. Statistical differences were tested using the log-rank test. Multivariate analysis was performed using Cox regression analysis. A *p* value of <0.05 was considered significant. The GraphPad v.5.0 (GraphPad Software Inc., San Diego, CA, USA) and MedCalc 11.2.0.0 (MedCalc Software, Ostend, Belgium) software were used for statistical analyses.

## 5. Conclusions

EBV^+^ and MSI gastric carcinomas are characterized by high PD-L1/PD-1 expression and abundant CD8^+^ tumor infiltrating lymphocytes. PD-L1 expression is not an independent prognostic factor.

## Figures and Tables

**Table 1:** Correlation between programmed cell death-ligand 1 (PD-L1) expression in tumor cells and clinic-pathological features.

Feature	Total Cases		EBV^+^ Cases		MSI Cases		MSS/EBV^−^ Cases	
	Total	PD-L1^+^	TOT	PD-L1^+^	TOT	PD-L1^+^	TOT	PD-L1^+^
Number of cases	169	31 (18)	33	15 (46) ***	59	14 (24) ***	77	2 (3) ***
Male	103 (61)	18 (58)	24 (73)	11 (73)	26 (44)	5 (36)	53 (69)	2 (100)
Female	66 (39)	13 (42)	9 (27)	4 (27)	33 (56)	9 (64)	24 (31)	0
Mean age years (range)	67 (36–90)	68 (51–84)	66 (36–86)	66 (51–86)	71 (49–90)	72 (51–84)	63 (38–84)	61 (52–70)
≥67 years	91 (54)	19 (61) ****	17 (52)	9 (60)	40 (68)	9 (64)	34 (44)	1 (50)
<67 years	78 (46)	12 (39) ****	16 (49)	6 (40)	19 (32)	5 (36)	43 (56)	1 (50)
Tumor location								
Cardias	9 (5)	3 (10)	4(12)	3 (20)	1 (2)	0	4 (5)	0
Body/fundus	38 (22)	8 (26)	14 (43)	6 (40)	8 (13)	2 (14)	16 (21)	0
Antrum	105 (62)	16 (51)	6 (18)	2 (13)	50 (85)	12 (86)	49 (64)	2 (100)
Stump	11 (7)	3 (10)	8 (24)	3 (20)	0	0	3 (4)	0
Others	6 (4)	1 (3)	1 (3)	1 (7)	0	0	5 (6)	0
Mean diameter mm (range)	52 (0–190)	60 (0–130)	52 (0–130)	52 (23–130)	61 (0–120)	61 (0–120)	45 (0–190)	47 (43–50)
≥52 mm	61 (36)	18 (58) ***	13 (39)	9 (60)	32 (54)	9 (64)	16 (21)	0
<52 mm	108 (64)	13 (42^)^ ***	20 (61)	6 (40)	27 (46)	5 (36)	61 (79)	2 (100)
Lauren classification								
Intestinal	118 (70)	26 (84)	26 (79)	12 (80)	48 (81)	12 (86)	44(57)	2 (100)
Diffuse	21 (12)	1 (3)	0	0	3 (5)	1 (7)	18 (23)	0
Indeterminate	30 (18)	4 (13)	7 (21)	3 (20)	8 (14)	1 (7)	15 (20)	0
Grading								
G1	10 (6)	1 (3)	3 (9)	1 (7)	3 (5)	0	4 (5)	0
G2	59 (35)	7 (23)	14 (42)	6 (40)	16 (27)	0	29 (38)	1 (50)
G3	87 (51)	22 (71) *	15 (46)	7 (46)	38 (64)	14 (100)	34 (44)	1 (50)
Not defined	13 (8)	1 (3)	1 (3)	1 (7)	2 (4)	0	10 (13)	0
Histotype-based prognostic classification								
Grade 1-low grade								
Muconodular	1 (1)	0	0	0	1 (2)	0	0	0
WD tubular	3 (2)	0	0	0	1 (2)	0	2 (3)	0
DD low grade	1 (1)	0	0	0	0	0	1 (1)	0
HLR	77 (45)	28 (90) ***	33 (100)	15 (100)	37 (63)	12 (86)	7 (9)	1 (50)
Grade 2-Intermediate grade								
Ordinary cohesive	58 (34)	2 (7)	0	0	16 (27)	1 (7)	42 (55)	1 (50)
Ordinary diffuse	18 (10)	0	0	0	0	0	18 (23)	0
Ordinary mucinous	3 (2)	0	0	0	1 (2)	0	2 (3)	0
Grade 3-High grade								
Anaplastic	5 (3)	1 (3)	0	0	1 (2)	1 (7)	4 (5)	0
Mucinous invasive	3 (2)	0	0	0	2 (3)	0	1 (1)	0
TNM Stage								
I	21 (12)	6 (19) *	11 (33)	5 (33)	5 (8)	1 (7)	5 (7)	0
II	65 (39)	16 (52) *	9 (27)	5 (33)	31 (53)	10 (71)	25 (32)	1 (55)
III	72 (43)	8 (26)	13 (40)	5 (33)	20 (34)	3 (22)	39 (51)	0
IV	9 (5)	1 (3)	0	0	2 (3)	0	7 (9)	1 (55)
No stage	2 (1)	0	0	0	1 (2)	0	1 (1)	0
pT								
T1a/b	7 (4)	4 (13) **	7 (21)	4 (27)	0	0	0	0
T2	25 (15)	4 (13) **	6 (18)	2 (13)	7 (12)	2 (14)	12 (16)	0
T3	93 (55)	20 (64)	17 (52)	8 (53)	39 (66)	11 (79)	37 (48)	1 (50)
T4a/b	44 (26)	3 (10)	3 (9)	1 (7)	13 (22)	1 (7)	28 (36)	1 (50)
pN								
N0	59 (35)	14 (45)	15 (46)	6 (40)	27 (47)	7 (50)	17 (22)	1 (50)
N1-3	108 (65)	17 (55)	18 (54)	9 (60)	31 (53)	7 (50)	59 (77)	1 (50)
CD8^+^ TILs > 9.5	107 (69)	29 (93) ***	33 (100)	15 (100)	42 (71)	12 (86)	32 (51)	2 (100)
CD8^+^ TILs ≤ 9.5	48 (31)	2 (7)	0	0	17 (29)	2 (14)	31 (49)	0

Legend. WD, well differentiated; DD, diffuse desmoplastic; HLR, high lymphoid response; * *p*-value < 0.05; ** *p*-value < 0.01; *** *p*-value < 0.001.

**Table 2:** Immunophenotype of TILs and PD-L1 expression on tumor and immune cells.

Feature	Total	EBV^+^ Cases	MSI Cases	MSS/EBV^−^ Cases	*p*
N. of cases	169	33	59	77	
PD-1^+^ cases	137 (81%)	30 (91%)	51 (86%)	56 (73%)	*
PD-1^+^ TILs Mean (range)	7 (0–74.6)	17.5 (0–74.6)	7 (0–35.3)	3.1 (0–26.7)	***
CD8^+^ TILs Mean (range)	28.9 (0–167.1)	64.2 (14.4–167.1)	23.1 (0–69.2)	15.8 (0–94.4)	*
PDL1^+^ TC cases	31 (18%)	15 (46%)	14 (24%)	2 (3%)	***
PDL1^+^ TC Mean % ^ (range)	25 (1–90)	33 (1–90)	18 (1–40)	17.5 (15–20)	**
PDL1^+^ IC cases	72 (42%)	31 (94%)	22 (37%)	19 (25%)	***
PDL1^+^ TI	31/72 (43%)	18/31 (58%)	12/22 (54%)	1/19 (5%)	***
PDL1^+^ IM	41/72 (57)	13/31 (42%)	10/22 (46%)	18/19 (95%)	

Legend. TC, tumor cells; IC, immune cells; TI, tumor infiltrating pattern; IM, invasive margin * *p*-value < 0.05; ** *p*-value < 0.01; *** *p*-value < 0.001; ^ in positive cases.

**Table 3:** Univariate analysis of survival probability.

Variable	*p*-Value	HR	95% CI
Intestinal type vs. others	0.0025	0.5156	0.3143–0.8457
HLR type vs. others	<0.0001	2.6683	1.7298–4.1160
Stage I/II vs. III/IV	<0.0001	2.9569	1.8946–4.6148
pT2/pT3 vs. pT4	<0.0001	2.6042	1.4849–4.5670
pN0 vs. pN1/pN2/pN3	0.0021	2.2075	1.4088–3.4592
CD8 > 9.5 cells/HPF	0.015	0.5637	0.3358–0.9462
EBV^+^/MSI vs. MSS/EBV^−^	0.003	1.8981	1.2207–2.9513
PD-L1 ≥ 5% vs. <5%	0.012	0.4102	0.2408–0.6988

Legend. HR hazard ratio; CI confidential interval.

**Table 4:** Multivariate analysis of survival probability.

Variable	Beta	SE	*p*-Value	Exp (Beta)	95% CI of Exp (Beta)
Tumor stage	0.9	0.2514	0.00035	2.4576	1.5052–4.0124
CD8 > 9.5 cells/HPF	0.55	0.2455	0.02455	1.7367	1.0760–2.8031
PD-L1 ≥ 5%	0.48	0.3864	0.2174	1.6105	0.7581–3.4211
Lauren classification	0.29	0.2616	0.2712	1.3335	0.8007–2.2208
EBV presence	0.29	0.4075	0.474	1.3388	0.6048–2.9636
MSI status	0.1	0.28	0.7271	1.1027	0.6387–1.9038

Legend. SE, standard error; CI confidential interval.

## Abbreviations

EBV	Epstein Barr Virus
MSI	Microsatellite instability
MSS	Microsatellite stable
TILs	tumor Infiltrating Lymphocytes
